# The Effect of Bornyl *cis*-4-Hydroxycinnamate on Melanoma Cell Apoptosis Is Associated with Mitochondrial Dysfunction and Endoplasmic Reticulum Stress

**DOI:** 10.3390/ijms19051370

**Published:** 2018-05-04

**Authors:** Tzu-Yen Yang, Yu-Jen Wu, Chi-I Chang, Chien-Chih Chiu, Mei-Li Wu

**Affiliations:** 1 Department of Food Science, National Pingtung University of Science and Technology, Pingtung 91202, Taiwan; gini0307@yahoo.com.tw; 2 Department of Beauty Science, Meiho University, Pingtung 91202, Taiwan; x00002180@meiho.edu.tw; 3 Department of Biological Science and Technology, Meiho University, Pingtung 91202, Taiwan; 4 Department of Biological Science and Technology, National Pingtung University of Science and Technology, Pingtung 91201, Taiwan; changchii@mail.npust.edu.tw; 5 Department of Biotechnology, Kaohsiung Medical University, Kaohsiung 80708, Taiwan; woodnettle2002@gmail.com

**Keywords:** melanoma, bornyl *cis*-4-hydroxycinnamate, proteomic, mitochondrial dysfunction, apoptosis, endoplasmic reticulum stress

## Abstract

Bornyl *cis*-4-hydroxycinnamate, an active compound isolated from *Piper betle* stems, was investigated in terms of its effects on A2058 and A375 melanoma cell proliferation and protein expression in this study. We used flow cytometric analysis to examine the early stages of apoptosis induced by bornyl *cis*-4-hydroxycinnamate in the two melanoma cell lines and employed comparative proteomic analysis to investigate the effects of this compound on protein expression in A375 cells. Master maps generated by PDQuest software from two-dimensional electrophoresis (2-DE) analysis of A375 cells showed that the expression levels of 35 proteins were significantly altered, with 18 proteins upregulated and 17 downregulated. The proteomics study identified several proteins that are involved in mitochondrial dysfunction and endoplasmic reticulum stress (ER stress), in addition to apoptosis-associated proteins, including prohibitin, hypoxia-upregulated protein 1, stress 70 protein, 78 kDa glucose-regulated protein (GRP78), and protein deglycase DJ-1 (protein DJ-1) in melanoma cells exposed to bornyl *cis*-4-hydroxycinnamate. The treatment also resulted in a marked decline of the mitochondrial membrane potential, in cytochrome *C* release into the cytosol, in the activation of Bcl-2-associated X protein (Bax), Bcl-2-associated death promoter protein (Bad), caspase-3, and caspase-9, and in the decreased expression of p-Bad, B-cell lymphoma 2 (Bcl-2), Bcl-xl, and induced myeloid leukemia cell differentiation protein-1 (Mcl-1), indicating that apoptosis induced by bornyl *cis*-4-hydroxycinnamate was mediated by the mitochondria through the caspase-dependent pathway. Also, salubrinal (an eukaryotic initiation factor 2α inhibitor; eIF2α inhibitor) was able to protect the cells from bornyl *cis*-4-hydroxycinnamate-induced apoptosis. Bornyl *cis*-4-hydroxycinnamate-related cell death also implied that the protein kinase R-like endoplasmic reticulum kinase (PERK)–eIF2α–ATF4–CHOP signal pathways was activated upon bornyl *cis*-4-hydroxycinnamate treatment. Altogether, our results support the conclusion that bornyl *cis*-4-hydroxycinnamate-induced apoptosis in melanoma cells is associated with mechanisms correlated with the activation of caspase cascades, mitochondrial dysfunction, and endoplasmic reticulum stress, and indicate that this molecule has the potential to be developed as a chemotherapeutic agent for human melanoma.

## 1. Introduction

Melanoma has a relatively low incidence among skin cancers, but also causes the vast majority of skin cancer deaths. In addition, the incidence rates of melanoma worldwide continue to rise rapidly [1]. In 2017, the American Cancer Society estimated that more than 87,000 new melanomas would be diagnosed in the US and approximately 10,000 people would die of melanoma that year [2]. The majority of patients with metastatic melanoma are almost invariably incurable because no effective chemotherapy or immunotherapy are available [3,4,5]. Therefore, currently, surgical resection alone or in combination with other therapies is still the best option to prolong the survival of patients [6,7]. Despite the great numbers of studies and clinical trials that have been performed in recent decades, dacarbazine, the most active single agent against melanoma, can only achieve an approximate 20% objective response rate [8,9], and interleukin (IL)-2 therapy only produces a response rate below 20% [10,11]. Therefore, the development of novel drugs and therapeutic approaches for malignant melanoma is an urgent medical issue.

*Piper betle* Linn. (Piperaceae), known as the betel vine, is a perennial semi-woody climber with a strong pungent and aromatic flavor that grows widely in Southeast Asia. It is an important medicinal and economical plant in some Asian countries, such as Taiwan and India, and has been proven to exhibit antioxidative, antimicrobial, and anti-hemolytic activities [12,13,14]. Several bioactive constituents such as lignins, polyphenols, alkaloids, steroids, saponins, tannins, and terpenes have been isolated from the leaves and stems of *P. betle* [15]. Bornyl *cis*-4-hydroxycinnamate is an active compound isolated from *P. betle* stem. In this study, we investigated the effects of bornyl *cis*-4-hydroxycinnamate on the proliferation of melanoma cells and employed a comparative proteomics approach to identify the molecules involved in bornyl *cis*-4-hydroxycinnamate-induced apoptosis in A2058 and A375 melanoma cells. We aimed to ascertain the underlying mechanism, which may lead to the development of bornyl *cis*-4-hydroxycinnamate as a new treatment agent or a potential strategy against human melanoma.

## 2. Results

### 2.1. Characterization of the Constituents of the Ethyl Acetate (EA) Fraction of P. betle Stems

**Figure 1:**
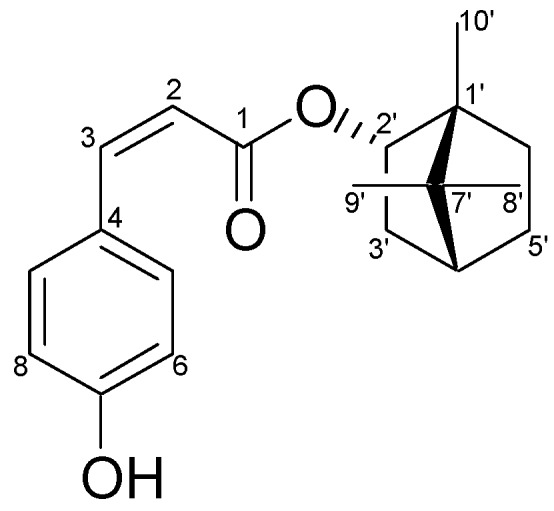
Chemical structure of bornyl *cis*-4-hydroxycinnamate isolated from the stem of *Piper betle.*

One monoterpene, bornyl *cis*-4-hydroxycinnamate, was afforded from the ethyl acetate fraction of *P. betle* stems, and its chemical structure was confirmed by comparing its physical and spectral data (specific rotation, mass spectrometry (MS), and nuclear magnetic resonance (NMR) spectroscopy) with the values described in the literature [16]. Bornyl *cis*-4-hydroxycinnamate (Figure 1): amorphous white powder; [α]^27^_D_ = +16.1° (c 0.5, CHCl_3_); ^1^H-NMR (400 MHz, CDCl_3_) δ 0.84 (3H, s, H-10′), 0.86 (3H, s, H-8′), 0.91 (3H, s, H-9′), 1.03 (1H, dd, *J* = 13.6, 3.2 Hz, H-3′b), 1.19 (1H, m, H-5′b), 1.24 (1H, m, H-6′b), 1.67 (1H, t, *J* = 4.4 Hz, H-4′), 1.72 (1H, m, H-5′a), 1.87 (1H, m, H-6′a), 2.41 (1H, m, H-3′a), 4.90 (1H, br d, *J* = 10 Hz, H-2′), 5.85 (1H, d, *J* = 12.8 Hz, H-2), 6.74 (2H, d, *J* = 8.4 Hz, H-6, H-8), 6.85 (1H, d, *J* = 12.8 Hz, H-3), 7.53 (2H, d, *J* = 8.8 Hz, H-5, H-9); ^13^C-NMR (CDCl_3_) δ: 13.5 (C-10′), 18.7 (C-9′), 19.6 (C-8′), 27.0 (C-6′), 27.9 (C-5′), 36.6 (C-3′), 44.8 (C-4′), 47.7 (C-7′), 48.7 (C-1′), 80.4 (C-2′), 115.2 (C-6, C-8), 117.0 (C-2), 126.8 (C-4), 132.0 (C-5, C-9), 144.0 (C-3), 157.3 (C-7), 168.0 (C-1); infrared spectroscopy (IR) (KBr) ν_max_: 3370, 1685, 1605, 1515, 1155 cm^−1^; electron-impact (EI) MS (70 eV) *m*/*z* (relative intensity) 300 [M]^+^ (10), 164 (5), 147 (100), 136 (3), 119 (20), 91 (15), 81 (6), 69 (3), 55 (4). NMR spectroscopy and mass spectrometry (MS).

### 2.2. Cytotoxic and Antiproliferative Effects of Bornyl cis-4-Hydroxycinnamate on Melanoma Cells

**Figure 2:**
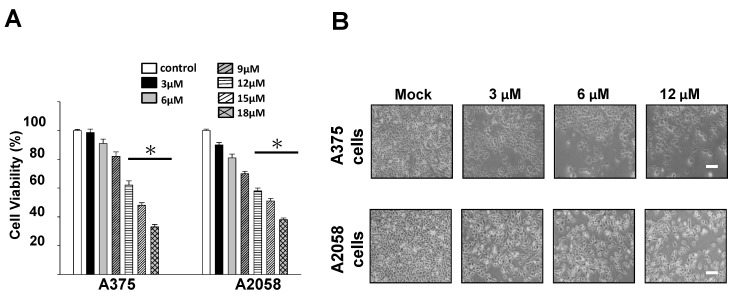
Evaluation of the cytotoxic and antiproliferative effects of bornyl *cis*-4-hydroxycinnamate on melanoma cells. (**A**) The cell viability of melanoma cells (A2058 and A375 cells) was inhibited in a concentration-dependent manner, as observed by MTT assay. Data are presented as mean ± SD. of at least three experiments independently. The results were analyzed with the statistical approach Student’s *t*-test (* *p* < 0.001, compared with the control). (**B**) Morphological changes and reduced cell populations of A2058 and A375 melanoma cells treated with different concentrations of bornyl *cis*-4-hydroxycinnamate (3, 6, and 12 μM). Scale bar: 50 μm.

To examine the potential cytotoxic effects of bornyl *cis*-4-hydroxycinnamate on melanoma cells (A2058 and A375 cells), 3-(4,5-Dimethylthiazol-2-yl)-2,5-diphenyltetrazolium bromide (MTT) assays and analyses of the morphological changes were performed. The cytotoxic effect of various concentrations of bornyl *cis*-4-hydroxycinnamate (3, 6, 9, 12, 15, and 18 µM) on A2058 and A375 melanoma cells was examined, and the results of the MTT assays revealed that bornyl *cis*-4-hydroxycinnamate reduced the proliferation of the two melanoma cell lines in a concentration-dependent manner (Figure 2A). With 12 µM bornyl *cis*-4-hydroxycinnamate treatment, the cell number of A2058 and A375 cells was significantly reduced (Figure 2B).

### 2.3. Apoptosis Is Triggered by Bornyl cis-4-Hydroxycinnamate in Melanoma Cells

**Figure 3:**
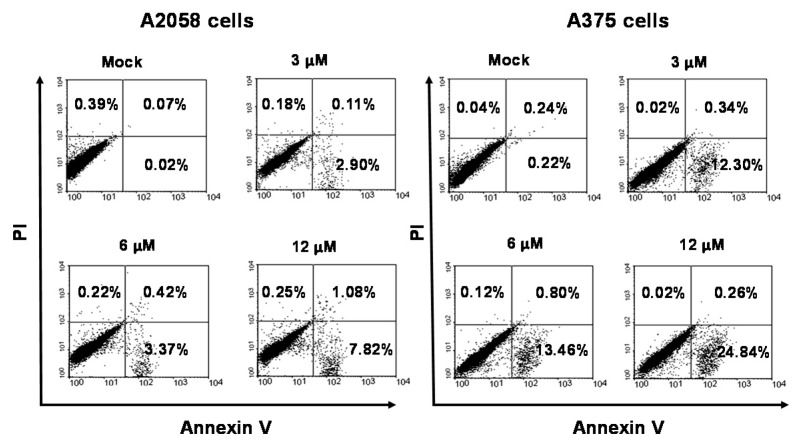
Bornyl *cis*-4-hydroxycinnamate-induced apoptosis in A2058 and A375 melanoma cells. Detection of apoptotic A2058 and A375 cells after bornyl *cis*-4-hydroxycinnamate treatment by flow cytometry based-annexin V-fluorescein isothiocyanate (FITC)/propidium iodide (PI) analysis.

Figure 2 shows that bornyl *cis*-4-hydroxycinnamate inhibited A2058 and A375 cell proliferation in a concentration-dependent manner. Apoptotic assays were then performed to investigate whether bornyl *cis*-4-hydroxycinnamate also induced melanoma cell apoptosis. After exposure to bornyl *cis*-4-hydroxycinnamate, flow cytometry analysis of annexin V-fluorescein isothiocyanate (FITC)/propidium iodide (PI) double stained cells showed that the mock treatment and the bornyl *cis*-4-hydroxycinnamate treatment at 3, 6, and 12 μM led A2058/A375 cells to display early apoptosis, at levels of 0.02%/0.22%, 2.9%/12.3%, 3.37%/13.46%, and 7.82%/24.84%, respectively (Figure 3). These results demonstrated that bornyl *cis*-4-hydroxycinnamate efficiently induced early apoptosis in A2058 and A375 melanoma cells.

### 2.4. Proteomic Analysis of Bornyl cis-4-Hydroxycinnamate-Treated A375 Cells

Following 12 μM bornyl *cis*-4-hydroxycinnamate treatment for 24 h, proteomic analysis was used to identify critical proteins involved in its cellular effect in A375 cells. Two-dimensional electrophoresis (2-DE) analysis with PDQuest image analysis software (Bio-Rad, Hercules, CA, USA) indicated that several proteins exhibited an intensity difference greater than 1.5-fold in the 2-DE maps. After gel digestion, liquid chromatograph (LC)-MS/MS analysis and MASCOT search engine v2.3 (Matrix Science, London, UK) protein identification demonstrated that a total of 35 differential protein spots were significantly altered between the control and the bornyl *cis*-4-hydroxycinnamate-treated A375 cells. In Table 1, the 35 identified proteins, with their detailed information, including calculated Mw (molecular weight)/pI (isoelectric point), MS/MS matched sequence coverage, MASCOT scores, fold changes (up-/downregulation), cellular location, and protein function, are presented. Among the 35 differentially expressed proteins, 18 were upregulated after bornyl *cis*-4-hydroxycinnamate treatment, i.e., nucleophosmin, hypoxia-upregulated protein 1, sodium/potassium-transporting ATPase subunit α-1, Hsp90, ATP synthase subunit β, vimentin, transitional endoplasmic reticulum ATPase (TER ATPase), proliferating cell nuclear antigen, tubulin β-4, prohibitin, glycogen phosphorylase, stress 70, fructose-bisphosphate aldolase A, glucose-regulated protein 78 (GRP78), glyceraldehyde-3-phosphate dehydrogenase (GAPDH), RNA-binding protein 8A, and Ran-specific GTPase-activating protein. On the other hand, 17 proteins were downregulated after bornyl *cis*-4-hydroxycinnamate treatment, i.e., voltage-dependent anion-selective channel protein, clathrin heavy chain 1, C-1-tetrahydrofolate synthase, serine hydroxymethyltransferase, poly(rC)-binding protein 2, protein DJ-1, prelamin-A/C, bifunctional UDP-*N*-acetylglucosamine 2-epimerase/*N*-acetylmannosamine kinase, α-enolase, elongation factor thermo unstable (EF-Tu), dihydrolipoyl dehydrogenase, heat-shock protein 105 kDa, tryptophan-tRNA ligase, triosephosphate isomerase, elongation factor 2 and 3-hydroxyacyl-CoA (coenzyme A) dehydrogenase type-2.

**Figure 4:**
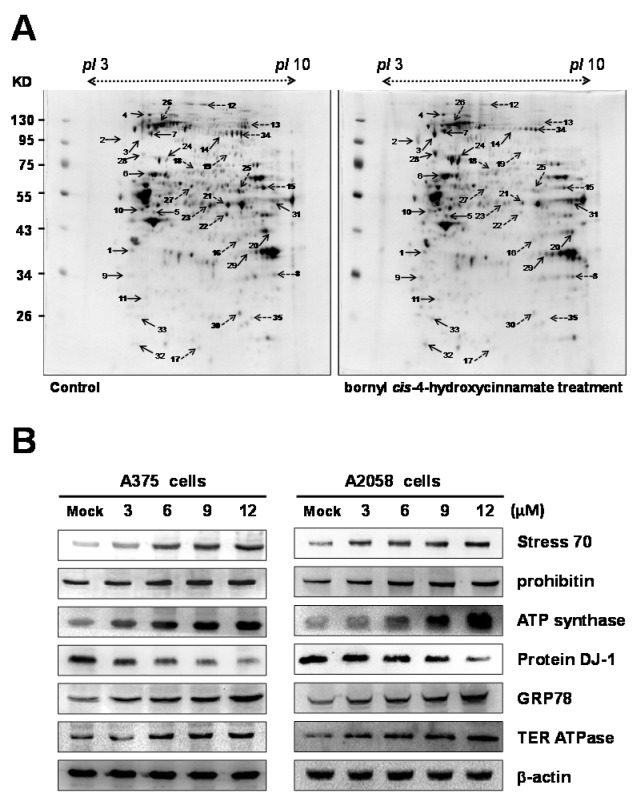
(**A**) The protein spots marked on 2-DE maps considered differentially expressed were identified by LC-MS/MS. The analysis was repeated three times. Isoelectric focusing (IEF) studies using 11-cm IPG strips (pI 3–10, Immobiline DryStrip). (**B**) Western blotting assay to validate the identified selected proteins from 2-DE, including stress 70, prohibitin, protein DJ-1, glucose-regulated protein 78 (GRP78), ATP synthase, and TER ATPase. Mock: control, dimethyl sulfoxide (DMSO)-treated cells. β-actin was used as the internal control. The solid arrows indicate upregulated proteins; the dashed arrows indicate downregulated proteins.

Differential proteins, such as stress 70, prohibitin, protein DJ-1, GRP78, ATP synthase, and TER ATPase were found to be associated with the induction of apoptosis and acted against cell proliferation. Western blotting was used to validate the changed profiles of these proteins. Altogether, proteomic analysis of 2-DE data and western blotting were in agreement in terms of the trends of changes of these identified proteins (Figure 4B).

### 2.5. Bornyl cis-4-Hydroxycinnamate Activates the Apoptosis Pathway through the Induction of Mitochondrial Depolarization

**Figure 5:**
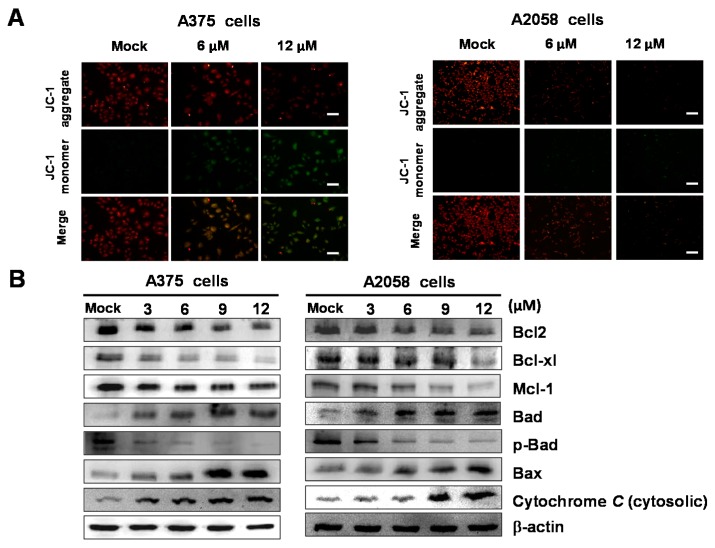
Bornyl *cis*-4-hydroxycinnamate induced apoptosis through mitochondria potential (Δψm) change and the mitochondrial-mediated pathway in A2058 and A375 melanoma cells. (**A**) A2058 and A375 melanoma cells were treated with or without bornyl *cis*-4-hydroxycinnamate; Δψm in melanoma cells was detected by JC-1 staining and analyzed using fluorescence microscopy. Scale bar: 50 μm. (**B**) Changes in Bcl-2, Bcl-xl, Mcl-1, Bad, p-Bad, Bax, and cytosolic cytochrome *C* expression in two melanoma cells treated with different concentrations of bornyl *cis*-4-hydroxycinnamate visualized by western blotting analysis. β-actin was used as the internal control.

As per the aforementioned results showing that bornyl *cis*-4-hydroxycinnamate induced apoptosis, we also noted that several mitochondrial-related proteins were identified in the bornyl *cis*-4-hydroxycinnamate-treated cells from 2-DE analysis, including prohibitin, stress 70, and ATP synthase subunit β. These proteins are known to be involved in energy production. We then employed the tetraethylbenzimidazolylcarbocyanine iodide (JC)-1 dye to measure the loss of mitochondrial membrane potential (Δψm) in A2058 and A375 cells after bornyl *cis*-4-hydroxycinnamate treatment. Fluorescence microscopy showed that bornyl *cis*-4-hydroxycinnamate-treated cells had a lower intensity of red fluorescence and a higher intensity of green florescence due to Δψm loss (Figure 5A). The mitochondrial-mediated apoptosis pathway plays an important role in apoptosis. We further analyzed molecules involved in the mitochondrial-mediated apoptosis pathway by examining the expression of related protein markers, including Bcl-2, Bcl-xl, Mcl-1, Bad, p-Bad, Bax, and cytosolic cytochrome *C*. As shown in Figure 5B, bornyl *cis*-4-hydroxycinnamate significantly increased the expression levels of Bad, Bax, and cytosolic cytochrome *C* in A2058 and A375 cells. In contrast, the levels of other marker proteins, such as Bcl-2, Bcl-xl, Mcl-1, and p-Bad, were decreased after bornyl *cis*-4-hydroxycinnamate treatment.

### 2.6. Bornyl cis-4-Hydroxycinnamate Activates the Caspase-Dependent Pathway Leading to Cell Apoptosis

**Figure 6:**
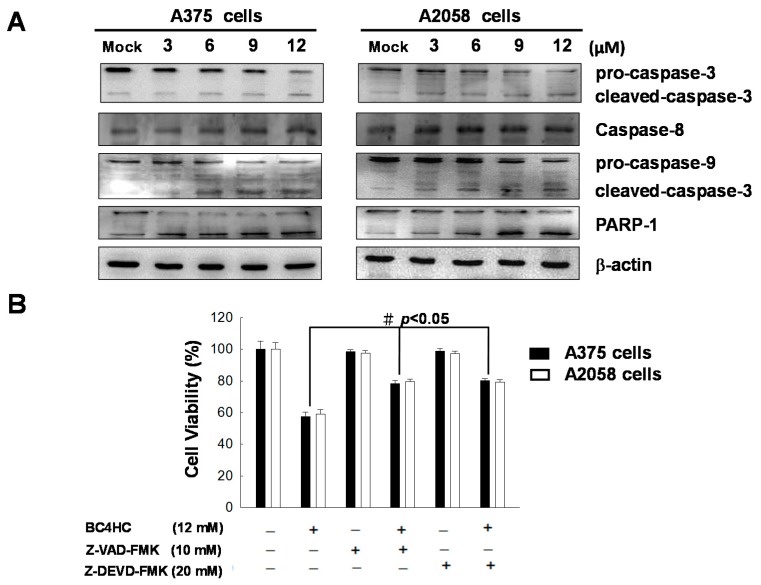
Bornyl *cis*-4-hydroxycinnamate induced apoptosis through caspase-dependent pathways. (**A**) The western blots show changes in apoptosis-associated protein expression levels in A2058 and A375 melanoma cells after treatment with bornyl *cis*-4-hydroxycinnamate. (**B**) Caspase-3 and caspase-9 inhibitors affected the viability of A2058 and A375 melanoma cells treated with bornyl *cis*-4-hydroxycinnamate. The cells were seeded onto a 24-well plate and pretreated with or without Z-DEVD-FMK (caspase-3 inhibitor) and Z-VAD-FMK (caspase-9 inhibitor), then treated with 12 µM bornyl *cis*-4-hydroxycinnamate. An MTT assay was performed for the evaluation of cell viability. Data are presented as mean ± SD. of at least three experiments independently. The results were analyzed with the statistical approach Student’s *t*-test (^#^ *p* < 0.05, compared with bornyl *cis*-4-hydroxycinnamate treatment groups). (BC4HC: bornyl *cis*-4-hydroxycinnamate).

To confirm the role of caspase in bornyl *cis*-4-hydroxycinnamate-induced apoptosis, the levels of caspase-3, caspase-8, caspase-9, and poly(ADP-ribose) polymerase (PARP)-1 were used to evaluate the status of caspase and its downstream effectors after bornyl *cis*-4-hydroxycinnamate treatment. As shown in Figure 6A, the expression of cleaved caspase-3 (17 kDa large subunit), cleaved caspase-9 (37 kDa large subunit), and cleaved PARP (89 kDa carboxyterminal catalytic domain) was increased in bornyl *cis*-4-hydroxycinnamate-treated A2058 and A375 melanoma cells, while the levels of pro-caspase-3 and pro-caspase-9 were decreased after bornyl *cis*-4-hydroxycinnamate treatment; the expression levels of caspase-8 were unchanged. These observations suggested that bornyl *cis*-4-hydroxycinnamate treatment activated the caspase-dependent pathway. To confirm whether the activation of the caspase cascade is crucial in bornyl *cis*-4-hydroxycinnamate-induced cell apoptosis, we used inhibitors against caspase-3 (Z-DEVD-FMK) and caspase-9 (Z-VAD-FMK). As shown in Figure 6B, cell death was suppressed in cells treated with either of these two inhibitors, suggesting that bornyl *cis*-4-hydroxycinnamate-induced apoptosis is mediated by caspase-3 and caspase-9 in melanoma cells.

### 2.7. Bornyl cis-4-Hydroxycinnamate Treatment Induces the Endoplasmic Reticulum (ER) Stress Pathway

**Figure 7:**
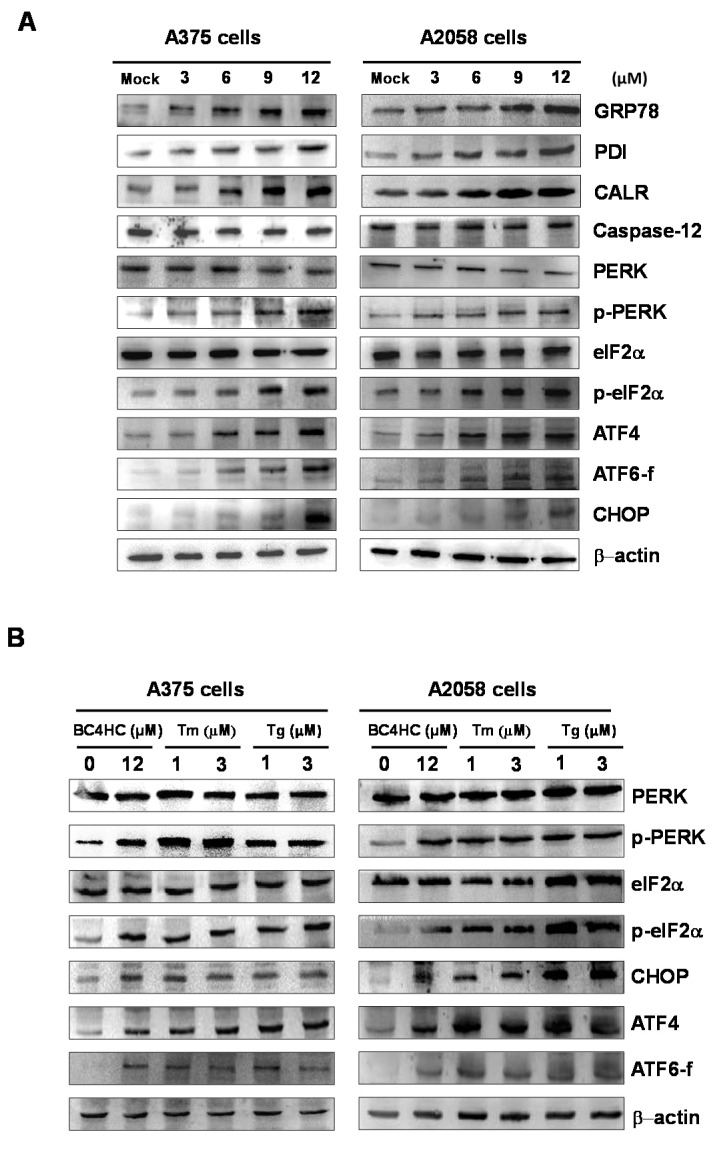
Expressions of endoplasmic reticulum (ER) stress-induced proteins in A375 and A2058 melanoma cells after bornyl *cis*-4-hydroxycinnamate treatment. (**A**) Expressions levels of ER stress response-related proteins in A2058 and A375 melanoma cells after bornyl *cis*-4-hydroxycinnamate treatment. (BC4HC: bornyl *cis*-4-hydroxycinnamate) (**B**) The increases in the expression levels of bornyl *cis*-4-hydroxycinnamate-induced ER stress-related protein were similar to those produced by the two ER stress-inducers agents, tunicamycin (Tm) and thapsigargin (Tg).

Next, we investigated whether the ER stress pathway was involved in bornyl *cis*-4-hydroxycinnamate-induced apoptosis in melanoma cells. Using western blot analysis, the expression levels of ER-related proteins GRP78 and TER ATPase were found to increase in the melanoma cells after bornyl *cis*-4-hydroxycinnamate treatment in a dose-dependent manner (Figure 4B). We then further verified the expression of three ER-resident transmembrane sensor proteins, i.e., endoribonuclease inositol-requiring enzyme 1α (IRE1α), protein kinase RNA-like endoplasmic reticulum kinase (PERK), and activating transcription factor 6 (ATF6), as well as of caspase-12 by western blot. The results showed that the expression of IRE1α and caspase-12 was unchanged after bornyl *cis*-4-hydroxycinnamate treatment. Under ER stress, endogenous ATF6 (p90 ATF6) is cleaved into a 50 KDa fragment transcription factor (p50 ATF6) and enters the nucleus to activate the *GRP78* genes [16]. Our results showed that p-PERK and p-eIF2α expression increased after bornyl *cis*-4-hydroxycinnamate treatment. The level of transcription factor ATF4, a downstream target of PERK in the PERK–eIF2α pathway, was also increased after treatment in A2058 and A375 melanoma cells. Additionally, the expression and nuclear translocation of ER stress-induced CCAAT/enhancer-binding protein (C/EBP)-homologous protein (CHOP) was also increased by bornyl *cis*-4-hydroxycinnamate treatment (Figure 7A).

Bornyl *cis*-4-hydroxycinnamate increased the ER stress-related protein expression levels similar to the two ER stress inducer agents tunicamycin (Tm) and thapsigargin (Tg), used to monitor the ER stress response. These results imply that bornyl *cis*-4-hydroxycinnamate-triggered cell apoptosis is mediated by the ER stress pathway in A2058 and A375 melanoma cells (Figure 7B).

**Figure 8:**
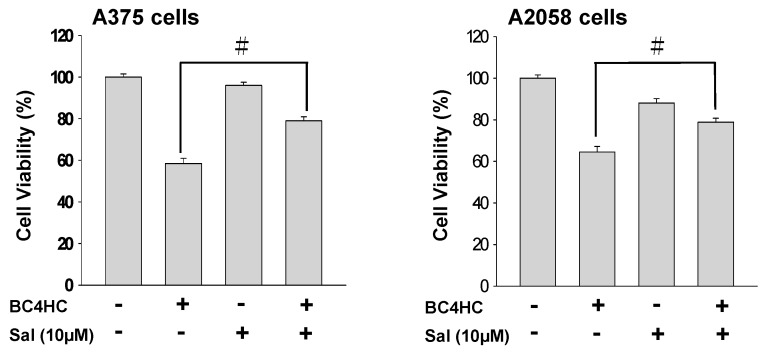
Cell survival after bornyl *cis*-4-hydroxycinnamate treatment was verified using the inhibitor salubrinal (Sal). The cells incubated with bornyl *cis*-4-hydroxycinnamate and salubrinal displayed increased survival compared to those treated with bornyl *cis*-4-hydroxycinnamate alone. Salubrinal treatment confirmed bornyl *cis*-4-hydroxycinnamate-induced apoptosis and inhibition of cell proliferation in melanoma cells. Data are presented as mean ± SD. of at least three experiments independently. The results were analyzed with the statistical approach Student’s *t*-test (^#^ *p* < 0.05, compared with BC4HC treatment groups).

To further demonstrate that bornyl *cis*-4-hydroxycinnamate-induced apoptosis occurs through ER stress-related pathways as described above, salubrinal (an inhibitor) was tested to examine PERK-activated cell apoptosis upon bornyl *cis*-4-hydroxycinnamate treatment. An increase in cell viability from 64% to 78% was observed in bornyl *cis*-4-hydroxycinnamate-treated A2058 and A375 cells following treatment with salubrinal at a concentration of 10 μM (Figure 8).

## 3. Discussion

In the current study, we investigated the cytotoxic effect of bornyl *cis*-4-hydroxycinnamate, isolated from *P. betel* stems, and studied the signaling pathways involved in the apoptosis induced by bornyl *cis*-4-hydroxycinnamate in melanoma cells. Our results demonstrate that bornyl *cis*-4-hydroxycinnamate inhibited cell proliferation (Figure 2) and increased early apoptosis rates (Figure 3) in A2058 and A375 melanoma cells, in a concentration-dependent manner. These findings support the conclusion that bornyl *cis*-4-hydroxycinnamate possesses activity against cell proliferation and may induce apoptosis in melanoma cells. 

The molecular mechanism involved in bornyl *cis*-4-hydroxycinnamate-induced apoptosis in A375 melanoma cells was elucidated from proteomic data, which showed significant changes in the expression of several crucial proteins, including sodium/potassium-transporting ATPase subunit α-1, ATP synthase subunit β, prohibitin, stress 70 protein, dihydrolipoyl dehydrogenase, and 3-hydroxyacyl-CoA dehydrogenase type-2, which are associated with mitochondrial function or apoptosis in melanoma cells. Prohibitin is located in the mitochondria and is known to regulate apoptosis, cellular signaling, cell migration, and cell proliferation and to stabilize mitochondria proteins [17,18,19,20]. The translocation of prohibitin to mitochondria together with p53 has been shown to be highly correlated with the suppression of cancer growth [21,22]. Therefore, the antiproliferative effects of bornyl *cis*-4-hydroxycinnamate are likely mediated by the mitochondria in A2058 and A375 cells and associated with the enhancement of prohibitin.

The apoptosis induced by bornyl *cis*-4-hydroxycinnamate is also a good indicator of the potential success of this molecule in the treatment of melanoma, as the development of anticancer drugs has often been based on the apoptosis effect as a potential mechanism of chemotherapy [23]. The mechanisms of apoptosis can be subdivided into two major pathways, i.e., the extrinsic and the intrinsic pathways. The former is also called the death receptor pathway, and the latter is also known as the mitochondrial pathway [24]. A study has suggested that the two pathways are related and influence each other [25], and molecules in the intrinsic pathway are located in either the ER or the mitochondria [26]. In addition, increased expression levels of the pro-apoptotic Bax and Bak proteins and decreased expression levels of the anti-apoptotic Bcl-2, Bcl-xl, and Mcl-1 proteins are known to play important roles in the intrinsic pathway and are associated with changes in the mitochondrial membrane potential and with the release of mitochondrial apoptotic factors [27]. The collapse of Δψm subsequently leads to cytochrome *C* release into the cytosol, resulting in caspase-9 activation, and further activates the downstream effector caspase-3. This process then causes poly(ADP-ribose) polymerase cleavage [28]. Existing information and our findings showing decreased Δψm and cytochrome *C* release from the mitochondria (Figure 5B) suggest that bornyl *cis*-4-hydroxycinnamate-induced apoptosis in A2058 and A375 cells occurs via the mitochondrial pathway. Additionally, the suppression of the anti-apoptotic Bcl-2, Bcl-xl, and Mcl-1 proteins and the augmented expression of the pro-apoptotic Bad and Bax proteins (Figure 5B), as well as the activation of caspase-3, caspase-9, and PARP (Figure 6), indicate that the caspase activation cascades are crucial in bornyl *cis*-4-hydroxycinnamate-induced apoptosis in A2058 and A375 cells. 

The ER plays many important roles in regulating essential cellular functions, including proper protein folding, protein synthesis, and calcium homeostasis [29]. When too many unfolded proteins accumulate in the ER, the homeostasis of the ER is destroyed, causing stress and leading to cells activating a self-rescue program or triggering apoptosis. In the presence of stress, the ER may react in various ways, including the induction of ER-associated degradation (ERAD), of the unfolded protein response (UPR), and of apoptosis [30,31]. Continued and severe ER stress increases the UPR, which will trigger apoptosis [32]. The UPR process is regulated by three ER signaling sensors, named protein kinase RNA-like endoplasmic reticulum kinase (PERK), inositol requiring enzyme 1-α (IRE1-α), and activating transcription factor 6 (ATF6). When unfolded proteins accumulate in the ER, chaperones, such as GRP78/GRP94, will release the transmembrane proteins IRE1-α, PERK, and ATF6, resulting in ER dysfunction. The PERK pathway either leads to cell survival controlled by autophagy [33] or to apoptosis by increasing ATF4/CHOP [34]. In the presence of ER stress, the cells activate a self-rescue program to trigger the UPR, which prevents cell death. Persistent ER stress initiates caspase-dependent apoptosis, leading to cell death [35]. When cells are under oxidative stress, unfolded or misfolded proteins accumulate in the ER, causing ER stress, and this imposed ER stress subsequently causes the UPR to alleviate the stress and restore ER homeostasis. The process upregulates chaperone GRP78 and disulfide isomerase protein, which promote protein folding and alleviate protein aggregation, and modulates calreticulin activity for the storing of calcium in the ER [36,37]. Our immunostaining results demonstrated that the levels of GRP78 and calreticulin were increased in the presence of a high concentration of bornyl *cis*-4-hydroxycinnamate, indicating that these proteins were activated to restore normal protein folding and reduce ER stress. If the UPR is insufficient to relieve cells from ER stress, the PERK, IRE1-α, and ATF6 pathways may activate downstream proteins to induce apoptosis in order to destroy the ER stress-damaged cells [38]. It was observed that PERK activates eIF2α, ATF4, and CHOP, causing apoptosis. The immunostaining results of this study also showed that the levels of p-PERK, p-eIF2α, and downstream ATF4 were increased, as was the expression of ATF6. These responses led to the activation of CHOP and increased its expression to induce apoptosis. The level of caspase-12 expression did not change, suggesting that the apoptotic mechanism in our study was mediated by the PERK–eIF2α–ATF4–CHOP signaling pathways in the ER (Figure 7A). In order to verify our hypothesis that bornyl *cis*-4-hydroxycinnamate-induced apoptosis in melanoma cells is associated with ER stress, we used ER stress agonists, namely, Tm and Tg (1 and 3 μM, respectively), to treat the cells. The results showed that the treatment with increasing concentrations of Tm and Tg resulted in trends of the expression of PERK, p-PERK, eIF2α, p-eIF2α, CHOP, ATF4, and ATF6 consistent with those of cells treated with bornyl *cis*-4-hydroxycinnamate. This finding suggests that bornyl *cis*-4-hydroxycinnamate-induced apoptosis occurs through a mechanism that involves ER stress (Figure 7B). We also used salubrinal, an eIF2α inhibitor, to further confirm our hypothesis. An MTT assay showed that the survival rate of cells treated with bornyl *cis*-4-hydroxycinnamate and salubrinal together was significantly higher than that of cells treated with bornyl *cis*-4-hydroxycinnamate alone (Figure 8). In conclusion, the results indicate that bornyl *cis*-4-hydroxycinnamate initiated ER stress and subsequently induced apoptosis in melanoma cells.

Overall, our results demonstrate that bornyl *cis*-4-hydroxycinnamate-induced apoptosis in A2058 and A375 cells is mediated by a dysfunction of the mitochondrial pathway, the activation of caspase cascades, and ER stress.

## 4. Materials and Methods

### 4.1. Reagents

Bornyl *cis*-4-hydroxycinnamate was isolated from *P. betel* stem and dissolved in DMSO at a concentration of 100 mM as a stock solution and stored at −20 °C. The compounds 3-(4,5-dimethylthiazol-2-yl)-2,5-diphenyltetrazolium bromide (MTT), Z-DEVD-FMK (caspase-3 inhibitor), Z-VAD-FMK (caspase-9 inhibitor), tunicamycin (Tm), thapsigargin (Tg), salubrinal, and dimethyl sulfoxide (DMSO), the protease inhibitor cocktail, and rabbit anti-human β-actin antibodies were purchased from Sigma (St Louis, MO, USA). The chemiluminescent horseradish peroxidase (HRP) substrate was obtained from Pierce (Rockford, IL, USA). Isoelectric focusing (IEF) strips and immobilized pH gradient (IPG) buffer were obtained from GE Healthcare (Buckinghamshire, UK). Dulbecco’s modified Eagle’s medium was obtained from Biowest (Nuaillé, France). An annexin V/FITC Apoptosis Detection kit was purchased from Pharmingen (San Diego, CA, USA). The cationic dye JC-1 was purchased from Biotium (Hayward, CA, USA). Rabbit anti-human cytochrome *C*, PARP-1, pro-casapse-3, cleaved caspase-3, pro-caspase-8, pro-caspase-9, cleaved caspase-9, caspase-12, Bax, Bad, p-Bad, Bcl-2, Bcl-xl, Mcl-1, PDI, calreticulin, ATP synthase, stress 70, GRP78, prohibitin, protein DJ-1, transitional endoplasmic reticulum ATPase, IRE1α, PERK, p-PERK, eIF2α, p-eIF2α, ATF4, ATF6-f, and CHOP antibodies were obtained from Cell Signaling Technology (Danvers, MA, USA). Goat anti-rabbit and horseradish peroxidase-conjugated IgG was purchased from Millipore (Bellerica, MA, USA).

### 4.2. General Instrumental Operation for the Isolation and Identification of Compounds

NMR spectra were analyzed in CDCl_3_ at room temperature using a Varian Mercury plus 400 NMR spectrometer, and the solvent resonance was used as the internal shift reference (tetramethyl silane [TMS] as standard). EI–MS were recorded on a SX-102A mass spectrometer (JEOL USA, Inc., Peabody, MA, USA). Thin-layer chromatography was used on silica gel 60 F254 plates (Merck KGaA, Darmstadt, Germany), and the spots were visualized by spraying with 10% H_2_SO_4_ solution. Silica gels (230–400 mesh ASTM, Merck KGaA) were used for column chromatography. Semi-preparative HPLC was performed using LiChrosorb Si 60 column, (7 μm, 250 × 10 mm; (Merck KGaA) on a LDC Analytical-III system. 

### 4.3. Source, Extraction, Fractionation, and Purification of P. betle Stem Compounds

Stems of *P. betle* were collected in Pingtung County, Taiwan. Air-dried stems of *P. betle* (3 kg) were ground using an electric high-speed grinder until a fine powder that passed through a 10-mesh sieve was obtained, and the powder was extracted with methanol (solid/solvent ratio = 1:5) at room temperature three times (7 d each). The filtrates were pooled and dried under reduced pressure to allow the solvent to evaporate. The dry crude residue was then suspended in water before being successively partitioned with ethyl acetate and *n*-butanol.

The ethyl acetate fraction (65 g) was separated into 24 fractions on a silica gel column (5 × 60 cm), using step-gradient elution with solvent mixtures of n-hexane and ethyl acetate of increasing polarity as eluents. Fraction 10 (4.2 g) was further chromatographed on a silica gel column (3 × 45 cm) using step-gradient elution with CH_2_Cl_2_–EtOAc (99:1 to 1:1) to give six fractions (each about 400 mL), 10 A–10 F. Semipreparative HPLC was used to separate Fr. 10 C by using *n*-hexane–CH_2_Cl_2_–EtOAc (16:6:1) to elute the sample (32.1 mg; Figure 1).

### 4.4. Cell Lines and Cell Culture Conditions

A2058 and A375 melanoma cells were obtained from the Food Industry Research and Development Institute (Hsinchu, Taiwan). A2058 and A375 melanoma cells were grown in Dulbecco’s modified Eagle’s medium (DMEM) supplemented with 10% (*v*/*v*) fetal bovine serum, 1 mM sodium pyruvate, 4 mM l-glutamine, 100 μg/mL streptomycin and 100 U/mL penicillin in a humidified incubator at 37 °C with 5% CO_2_.

### 4.5. Cytotoxicity Assessment

A2058 and A375 cells were seeded in 96-well plates at a density of 1 × 10^5^/well with 200 μL of Dulbecco’s modified Eagle medium (DMEM) and 10% fetal bovine serum in a humidified, 5% CO_2_ atmosphere at 37 °C. The cells in each well were treated with bornyl *cis*-4-hydroxycinnamate (at concentrations of 0, 3, 6, 9, 12, 15, and 18 μM) for 24 h. Cells treated with DMSO without bornyl *cis*-4-hydroxycinnamate were used as a control. After a 24 h incubation period, 50 μL of MTT (0.5 mg/mL stock) was added to the cells, followed by incubation for 2 h. The medium was then removed, and DMSO (200 μL) was added to dissolve the formazan. The optical density (OD) was determined at 595 nm on an ELISA reader (Bio-Rad, Hercules, CA, USA) to provide a relative estimate of cell viability. All experiments were performed in at least triplicate to confirm their reproducibility. 

### 4.6. Flow Cytometric Analysis

To analyze apoptosis in A2058 and A375 melanoma cells after bornyl *cis*-4-hydroxycinnamate treatment, the cells were stained with an annexin V-FITC apoptosis staining kit (Pharmingen, San Diego, CA, USA) and analyzed as described in previous reports [39,40]. Briefly, 1 × 10^6^ cells were seeded into 5 cm Petri dishes and treated with different concentrations of bornyl *cis*-4-hydroxycinnamate (3, 6, and 12 μM) for 24 h. Then, the cells were harvested and stained with annexin V-FITC/propidium iodide (PI) in a cell culture incubator for 30 min. Samples were then assessed using a FACScan flow cytometer (Becton-Dickinson, Mansfield, MA, USA), and the data were analyzed using FlowJo software v10.4.2 (TreeStar, Inc., Ashland, OR, USA). 

### 4.7. Protein Lysate Preparation

After bornyl *cis*-4-hydroxycinnamate treatment (0, 3, 6, 9 and 12 μM) for 24 h, A2058 and A375 cells were lysed with a cell extraction buffer (BioSource International, Camarillo, CA, USA). The supernatants were quantified using a Bio-Rad protein assay for sodium dodecyl sulfate polyacrylamide gel electrophoresis (SDS-PAGE). In addition, some of each supernatant was precipitated using a 10% cold trichloroacetic acid (TCA)/acetone solution containing 20 mM dithiothreitol (DTT) overnight at −20 °C. The protein pellet was dissolved in a rehydration buffer (2 M thiourea, 6 M urea, 20 mM DTT, 0.5% IPG buffer, 0.5% 3-[(3-cholamidopropyl)dimethylammonio]-1-propanesulfonate (CHAPS), and 0.002% bromophenol blue) at 4 °C, and the protein concentrations were determined using a 2-D Quant Kit (GE Healthcare) for 2-DE analysis.

### 4.8. Two-Dimensional Gel Electrophoresis and Differential Proteomic Analyses

For two-dimensional gel electrophoresis (2-DE), protein samples (200 μg) from the control cells and the cells treated with 12 μM bornyl *cis*-4-hydroxycinnamate were analyzed using a GE Healthcare Ettan IPGphor 3. For second-dimension electrophoresis, the equilibrated strip was placed onto the top of a 12.5% SDS-PAGE gel and run on an SE 600 Ruby Vertical electrophoresis system (Hoefer, Holliston, MA, USA). Each sample was run in triplicate, and the spots on the 2-DE gels were visualized by silver staining. The gels were scanned and then analyzed using PDQuest image analysis software v8.0.1 (Bio-Rad). Each triplicate sample was normalized prior to statistical analysis. The protein spots with an intensity difference between the control and bornyl *cis*-4-hydroxycinnamate-treated cells greater than 1.5-fold were identified, and statistically significant differences in 2-DE between 11-epi-sinulariolide acetate-treated HA22T cells and the control [41] were identified. The protein spots of interest were excised from the 2-DE gels, and in-gel digestion was performed with trypsin. The digested samples were analyzed by LC/MS/MS using a QTRAP 5500Q mass spectrometer (AB Sciex, Framingham, MA, USA). MS scanning ranged from *m*/*z* 100 to1000. The raw MS data were converted into the text file format WIFF using Analyst 1.5.1 (AB Sciex, Framingham, MA, USA).

### 4.9. Assessment of the Mitochondrial Membrane Potential (Δψm)

The mitochondrial membrane potential (Δψm) was determined using cells stained with the cationic dye JC-1. Briefly, A2058 and A375 cells at 1 × 10^5^ cells/well in a 12-well plate were treated with different concentrations of bornyl *cis*-4-hydroxycinnamate (0, 3, 6, and 12 μM). The treated cells were collected and washed twice with PBS, incubated with 70 μL of JC-1 staining solution, and placed in a cell culture incubator for 30 min. Following washing with a buffer, the cells were then directly observed under a fluorescence microscope [42].

### 4.10. Antibody and Western Blot Analysis

Western blot analysis was performed according to previous reports. The primary antibodies used were: anti-human stress 70, GRP78, ATP synthase, prohibitin, protein DJ-1, Transitional endoplasmic reticulum ATPase, Bax, Bad, p-Bad, Mcl-1, Bcl-2, Bcl-xl, PARP-1, pro-caspase-3, cleaved-caspase-3, caspase-8, pro-caspase-9, cleaved-caspase-9, cytochrome *C*, PDI, calreticulin, IRE1α, caspase-12, PERK, p-PERK, elf2α, p-eIF2α, ATF4, ATF6-f, CHOP, and β-actin antibodies. Secondary antibodies (horseradish peroxidase-conjugated goat anti-rabbit, 1:5000 in blocking solution) were added, followed by an incubation for 2 h. The signals were visualized using a chemiluminesence detection kit (Pierce Biotechnology, Rockford, IL, USA).

### 4.11. Statistical Analysis

All experiments were repeated at least in triplicate. Data analysis was performed using Student’s *t* test (Sigma-Stat 2.0, San Rafael, CA, USA); *p* values < 0.05 were considered significant.

## 5. Conclusions

**Figure 9:**
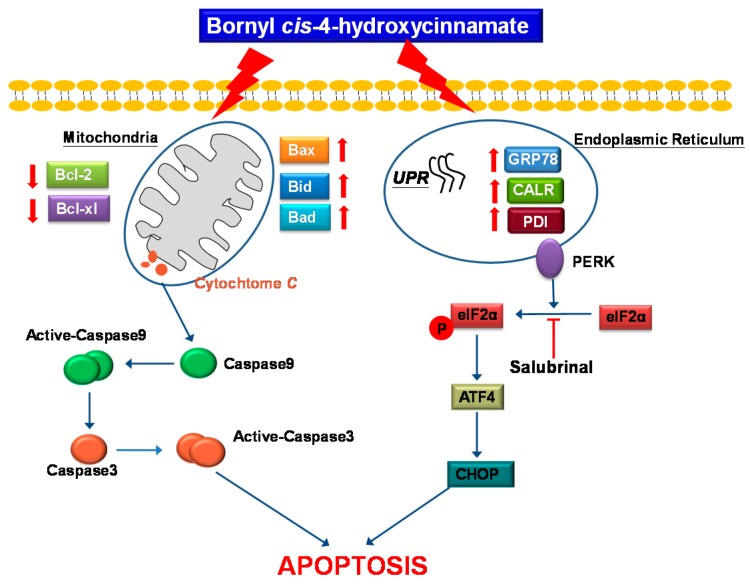
Bornyl *cis*-4-hydroxycinnamate-induced apoptotic pathway in A2058 and A375 melanoma cells. The anti-ancer effect of bornyl *cis*-4-hydroxycinnamate is mediated by the induction of mitochondria dysfunction and ER stress signaling pathways.

Our results established that bornyl *cis*-4-hydroxycinnamate extracted from *P. betel* stems possesses the ability to induce apoptosis in A2058 and A375 melanoma cells. A differential proteomic analysis identified several important mitochondrial and endoplasmic reticulum proteins and proved that mitochondrial dysfunction and the activation of endoplasmic reticulum stress pathways are key molecular mechanisms in the apoptosis induced by bornyl *cis*-4-hydroxycinnamate (Figure 9). Our findings provide evidence that bornyl *cis*-4-hydroxycinnamate has the potential to be developed as new therapeutic agent for melanoma treatment.

## Figures and Tables

**Table 1:** The regulation (fold changes) of the differentially expressed proteins refers to a 24 h treatment with bornyl *cis*-4-hydroxycinnamate.

Spot No.	Protein Name	Accession. No.	Mw/pI	Peptide Matched	Sequence Covered (%)	MASCOT Score	Regulation (Fold-Change)	Cellular Component	Protein Function
1	Nucleophosmin	P06748	32.72/4.64	40	30	1737	+1.53	Nucleus	Regulation of ARF–p53 tumor suppressor pathway
2	Sodium/potassium-transporting ATPase subunit α-1	P05023	114.15/5.33	47	28	2125	+2.76	Mitochondrion	Sodium/potassium-exchanging ATPase activity
3	Hsp90	P08238	83.56/4.97	113	49	3086	+2.74	Cytoplasm	Protein folding
4	Hypoxia upregulated protein 1 (heat shock protein 70 family)	Q9Y4L1	111.49/5.16	6	6	52	+2.21	Endoplasmic reticulum	Stress response
5	ATP synthase subunit β	P06576	56.52/5.26	51	57	1865	+2.89	Mitochondrion	Mitochondrial membrane ATP synthase
6	Vimentin	P08670	53.67/5.06	5	9	58	+2.72	Cytoplasm	Cytoskeletal protein
7	Transitional endoplasmic reticulum ATPase	P55072	89.96/5.14	29	20	432	+3.12	Endoplasmic reticulum	Involved in the formation of the transitional endoplasmic reticulum (tER)
8	Voltage-dependent anion-selective channel protein	P21796	30.87/8.62	42	68	1261	−1.55	Cytoplasm	Involved in cell volume regulation and apoptosis
9	Proliferating cell nuclear antigen	P12004	29.09/4.57	61	73	1246	+1.36	Nucleus	DNA repair and damage
10	Tubulin β-4B	P68371	50.26/4.79	27	40	601	+3.4	Cytoplasm	Structural constituent of cytoskeleton
11	Prohibitin	P35232	29.84/5.57	73	78	2345	+1.51	Mitochondrion	Inhibits DNA synthesis
12	Clathrin heavy chain 1	Q00610	193.29/5.48	41	29	1104	−1.51	Cytoplasm	Intracellular trafficking
13	C-1-tetrahydrofolate synthase	P11586	102.19/6.89	6	6	54	−1.56	Cytoplasm	Hydrolase
14	Glycogen phosphorylase	P11216	97.33/6.40	43	44	1331	+1.59	Mitochondrion	Glycosyltransferase
15	Serine hydroxymethyltransferase	P34897	56.42/8.76	22	18	198	−2.11	Mitochondrion	Associates with mitochondrial DNA
16	Poly(rC)-binding protein 2	P40555	37.99/6.66	30	28	491	−1.52	Cytoplasm	Chaperone
17	Protein DJ-1	Q99497	20.05/6.33	56	68	2434	−3.21	Endoplasmic Reticulum	Protects cells against oxidative stress and cell death
18	Prelamin-A/C	P02545	74.38/6.57	67	48	2452	−3.13	Nucleus	Structural molecule activity
19	Bifunctional UDP-*N*-acetylglucosamine 2-epimerase/*N*-acetylmannosamine kinase	Q9Y223	80.21/6.32	3	2	102	−1.52	Cytoplasm	Regulates and initiates biosynthesis of *N*-acetylneuraminic acid
20	Fructose-bisphosphate aldolase A	P04075	39.85/8.30	6	10	121	+3.25	Cytoplasm	Plays a key role in glycolysis and gluconeogenesis
21	α-enolase	P06733	47.48/7.01	355	75	8369	−1.81	Cytoplasm	Multifunctional enzyme (Transcription regulation)
22	Elongation factor Tu,	P49411	49.85/7.26	201	69	5544	−2.36	Mitochondrion	Elongation factor
23	α-enolase	P06733	47.48/7.01	143	76	3720	−2.94	Cytoplasm	Lyase
24	Stress-70 protein	P38646	73.92/5.87	432	72	11,267	+3.15	Mitochondrion	Chaperone
25	Dihydrolipoyl dehydrogenase, mitochondrial	P09622	54.72/7.95	28	27	630	−2.73	Mitochondrion	Oxidoreductase
26	Heat-shock protein 105 kDa	Q92598	97.73/5.28	120	60	2474	−1.52	Cytoplasm	Response to unfolded protein
27	Tryptophan-tRNA ligase	P23381	53.48/5.83	57	52	1802	−1.53	Cytoplasm	ATP binding
28	GRP78	P11021	72.40/2.07	49	53	1322	+3.23	Endoplasmic reticulum	Stress response
29	Glyceraldehyde-3-phosphate dehydrogenase (GAPDH)	P04406	36.20/8.57	88	84	1998	+1.54	Cytoplasm	Assembly of the cytoskeleton
30	Triosephosphate isomerase	P60174	31.06/5.65	54	70	1494	−2.78	Cytoplasm	Triose-phosphate isomerase activity
31	ATP synthase subunit β	P06576	56.52/5.26	23	37	524	+2.29	Mitochondrion	Mitochondrial membrane ATP synthase
32	RNA-binding protein 8A	Q9Y5S9	19.93/5.50	15	33	348	+1.57	Nucleus	mRNA binding
33	Ran-specific GTPase-activating protein	P43487	23.47/5.19	24	54	484	+1.52	Cytoplasm	GTPase activation
34	Elongation factor 2	P13639	96.26/6.41	175	58	2493	−1.51	Cytoplasm	GTPase activity
35	3-hydroxyacyl-CoA dehydrogenase type-2	Q99714	27.13/7.66	59	86	1770	−1.55	Mitochondrion	Mitochondrial tRNA maturation
